# The Rights of *Children on the Move* and the Budapest Declaration

**DOI:** 10.3390/children5050061

**Published:** 2018-05-17

**Authors:** Charles Oberg

**Affiliations:** Divisions of Global Pediatrics and Epidemiology & Community Health, University of Minnesota, 717 Delaware Street, Minneapolis, MN 55414, USA; oberg001@umn.edu

**Keywords:** migration, refugee, internally displaced persons (IDP), immigrant, children’s rights, Budapest Declaration

## Abstract

It has been estimated that more than 50,000,000 children and youth have migrated across borders or been forcibly displaced within their own country. They consist of refugees, asylum seekers, internally displaced persons (IDP), economic migrants, and exploited trafficked children. They are virtually “stateless”, children deprived of the protective structures of state and family that they need and deserve and unrecognized by either their country of origin or the international community. This opinion piece starts with the personal reflections of its author on his recent work in Middle East refugee camps. It then explores the prevalence and demographics of these children and their plight. It examines the United Nation’s Convention on the Rights of the Child (CRC) and other international conventions designed to protect them. It also summarizes the International Society of Social Pediatrics and Child Health (ISSOP) Budapest Declaration on the Rights, Health and Well-Being of Children and Youth on the Move as a framework for improved care and vehicle for change.

## 1. Introduction

It has been estimated that more than 50,000,000 children have migrated across borders or been forcibly displaced within their own country [1]. This includes 28,000,000 uprooted children who are either refugees living outside their country of origin or internally displaced due to wars, violence and conflict [2]. What is even more staggering is the 75% increase in the number of child refugees between 2010 and 2015 [3].

This crisis now directly affects every continent on the globe and indirectly each and every one of us. Over the last few years, the plight of children on the move became personal. I had the opportunity to work in several refugee camps serving displaced children. The first was on the Greek island of Lesvos. I volunteered with the Boat Refugee Foundation (BRF), a Netherlands based non-governmental organization NGO. The majority of my time was spent in the notorious Moria Refugee Camp with over 4500 refugees. Based in an old army compound, the camp was defined by the steel gates, high fencing and barbwire on the perimeter with an amorphous sea of tarps and tents on the inside. It was during the winter months and a few days prior to my arrival, the weather had turned brutally cold. Over a foot of snow was followed by freezing rain. The cold and dampness penetrated to the bone. Food queues, inadequate unsanitary toilet facilities and ubiquitous garbage were the norm. More recently, I traveled with the Syrian American Refugee Society (SAMS) to provide care to Syrian refugee children in multiple locations in northern Jordan. The most were in Al Zaatari, presently the second largest refugee camp in the world with over 80,000 inhabitants.

Despite best efforts, primary care was inadequate, referrals limited or non-existent and quality shelter and nutrition hard to provide in such settings. Children spend days, weeks and months with little opportunity to play in a safe and nurturing environment. There are multiple NGO's providing assistance including UNHCR, Save the Children, Euro Relief, the Red Cross/Red Crescent and others. However, the task of providing a safe milieu to grow, thrive and play is limited. In addition, many of the children have experience and/or witnessed trauma either in their home country or on their exodus to safety and a new life. Access to mental health services is woefully limited. Almost all the children and youth had experienced trauma. Some beaten, shot, tortured, and raped and all had experienced the stress of living in unlivable conditions. The complaints were a blur of physical, mental, and spiritual aliments.

Yet there was a palpable hope that one-day things would be better with aspirations of a better future. Daily they expressed their gratitude that someone would listen as they shared the story of their journey, affirmed their worth, acknowledged their struggle and celebrated their humanity.You could see it in their eyes and their smiles that each was seeking a better life for themselves and their children. I saw no terrorist. I just saw families, children, men and women—all vulnerable and suffering.

The children I saw were just a fraction of the millions of children and youth who have been displaced from their homes due to war, conflict, natural disasters and families seeking a better life.

## 2. The Scope of the Problem

The problem of children displaced from their homes spans decades and will most certainly worsen in the future due to the scope of the problem and the lack of an appropriate international response. This section provides a sampling of the magnitude of the problem. It is essential to first identify that the plight of Palestinians represent the largest and longest displaced group of refugees. For seven decades the global community has closed its eyes and has ignored millions of forgotten children now living in the occupied territories of Gaza and the West Bank as well as those who been dispersed throughout the Middle East in Lebanon, Jordan, and Syria. The majority of Palestinians (3.7 million children and young people) are under the age of 25, and 40% of (2.5 million) are under the age of 18 [4]. Every decade since has experienced its own diaspora. This includes but is not limited to the exodus that started from Southeast Asia in the 1970–1980’s and Somalia in the 1990’s [5,6]. Since the turn of the millennium, refugees have fled the conflict and wars in Iraq, Afghanistan, and Syria. Africa has also experienced multiple displacements of children throughout the continent. Most recently the world has witnessed the persecuted Rohingya fleeing Myanmar into Bangladesh [7,8].

Children on the move also include millions of children migrating either with their families or as unaccompanied children and youth who are not fleeing war but are seeking a safer environment and opportunity for the future. Frequently labeled economic migrants, they are trying to escape poverty, exploitation, limited opportunity and frequently violence [9]. This includes the children and youth being forced into child labor or sex trafficked [10,11].

The situation has been exacerbated by a gradual shift in our response to this crisis at the national and international level. Though not uniform, we see growing intolerance and disdain translated into international policy. A growing fervor of fear and xenophobia has led to the closing of borders, stricter application of refugee and asylum criteria and increasing deportation, all of which are intended to stop the human flow seeking safety and freedom. The result is “stateless” children who are neither welcomed from their country of origin or the country where their exodus ends [12]. Do these children have rights and if so what moral obligations do we, as a global community, have in addressing their plight?

## 3. Children’s Rights and International Protection

In 1923, Eglantyne Jebb, who witnessed and documented the devastating effects of World War I on European children, penned a *Declaration of the Rights of the Child.* She later went on to start the Save the Children dedicating her life to children displaced by tragedy and war [13]. The Declaration was subsequently adopted by the United Nations in 1952 [14]. 

The Declaration espoused the basic principle of civil and political as well as economic, societal and cultural rights for all children. To put the Declaration into force, the UN General Assembly passed the UN Convention on the Rights of the Child on 20 November 1989 and has become the most universally accepted treaty in the history of the United Nation [15]. Globally every nation has signed the CRC (UN Convention on the Rights of the Child) except for the United States and South Sudan. The Convention establishes the responsibility of governments, institutions, citizens and families to ensure that the rights of the child are respected and all actions are directed toward achieving the “best interest of the child” [16].

The Convention provides a framework for the protection of children on the move. It is of particular concern that most professionals who deal with families with children are unfamiliar with the provisions of the UN Convention on the Rights of the Child (CRC). The UN CRC was the first legally binding international document to recognize the rights of the child. It provides a firm legal, moral, and ethical basis to achieve equity in response to children on the move [17].

Two other UN Conventions speak to the need for vulnerable children on the move. The first is the United Nations Convention relating to the Status of Refugees, which was adopted in 1951 and entered into force in 1954. It remains the centerpiece of international refugee protection today [18]. The second is the 1990 International Convention on the Protection of the Rights of All Migrant Workers and Members of their Families, which reiterates the principle of universal protection [19].

## 4. Budapest Declaration

In November 2017, the International Society of Social Pediatrics and Child Health (ISSOP) passed the *Budapest Declaration—On the Rights, Health and Well-Being of Children and Youth on the Move*. This society of international pediatricians was aware of the unprecedented global displacement of children and its perverse effects on their health and well-being [20]. The Declaration advocates for the following provisions:

- Entitles all children to the full complement of rights—regardless of their displacement status;
- Enumerates requirements for a holistic response to their physical and mental health risks and needs;
- Defines the elements of leadership for pediatricians and organizations;
- Outlines requirements for evidenced-base policies, protocols and evaluation;
- Grounds this work in a global context;
- Establishes the structure of a *Child Health Action Plan for Children and Youth on the Move*.

In addition, cognizant of violations of human rights resulting from displacement, the *Declaration* establishes the first comprehensive child rights-based blueprint for global pediatric leadership and action that integrates clinical care, systems development and public policy [21]. Specifically, in the domain of clinical care it recommends; health assessments and continuity of care should be performed in a manner that is sensitive to their cultural and ethnic origins. Specifically, an awareness that refugees and immigrant families are not a homogenous population. 

Rather a heterogeneous tapestry of different cultures with different beliefs around health, wellness and approaches to care. In addition, that even within a specific region or country numerous languages, dialects and distinct ethnic backgrounds must be acknowledged as providers seek to establish trust and a meaningful healing relationship. It must take place with informed consent and include participation in physical and mental health care decision making. In addition, professionals working with children should be trained in cultural and linguistic competency and how to work with interpreters. 

Finally, care delivery must incorporate a trauma-informed approach to care. Trauma-informed approaches must assure that the care delivered is aware and sensitive to past traumas that have occurred in the child’s home country, during their journey and in the new location that they have arrived. All efforts must be made to not re-traumatize the family or children in the administration of care. It must also establish therapeutic approaches that are developmentally appropriate for the children and youth who are being served with ancillary strategies and complementary approaches that promote healing. Health systems must recognize the physical and mental vulnerabilities across the age span including pregnant women newborns, children, adolescents and young adults.

In addition, the needs of children with disabilities must also be addressed. Service delivery systems for these children and youth, even in countries with well-established health systems, must address the fragmentation and barriers to optimal care. 

An essential element of new policies is that every nation state should advance *Health in all Policies* with a commitment to global public heath that advances equity for all within their borders [22]. States parties should be held accountable for their actions to children and youth on the move within their boundaries to ensure the full implementation of the rights articulated in the CRC, and this accountability should be addressed in their periodic reports to the Committee on the Rights of the Child. As of May 2018, fifteen International Pediatric Professional Associations have endorsed the Budapest Declaration representing pediatricians from North and South America, Europe, Asia and Australia.

## 5. Conclusion

Pediatricians and all professionals dedicated to the care of children have critical roles to play in response to the unprecedented global displacement of children. Pediatric leadership is essential and will consist of clearly delineating roles and responsibilities for engagement with other global organizations seeking change. This must include cooperation with key partners such as the UN Childen’s Fund (UNICEF), the World Health Organization (WHO) and the UN High Commissioner for Refugees (UNHCR). It will also include participation in a larger coalition of other NGO’s whose goals align including the International Organization for Migration (IOM), Save the Children, and Initiative for Child Rights in the Global Compact. As the world seeks to close international borders and to implement travel bans I make a plea for benevolence and tolerance. We must find policy solutions and concrete programmatic change to address the needs of children and youth on the move. To that end ISSOP has formulated a *Child Health Action Plan for Children and Youth on the Move* and has convened a workgroup to undertake its implementation. It presently consists of twenty-five child health professionals from fifteen countries. Together with other professional organizations and NGO’s an effort will be made to affect positive change. The effort will attempt to shift the global response from restricting access to expansion and openness. In the end let us remember that our kindness will make us safer than fear. This effort is part of a larger global awareness. Presently, the United Nations has undertaken a dialogue and initiated negotiations concerning Global Compact on Refugees and Migrants. It is possible that states are awakening to the particular vulnerabilities of children and youth on the move [23]. 
